# Correlation of Altered Expression of the Autophagy Marker LC3B with Poor Prognosis in Astrocytoma

**DOI:** 10.1155/2014/723176

**Published:** 2014-05-12

**Authors:** Daniel Winardi, Hung-Pei Tsai, Chee-Yin Chai, Chia-Li Chung, Joon-Khim Loh, Yung-Hsiang Chen, Ching-Liang Hsieh

**Affiliations:** ^1^Graduate Institute of Integrated Medicine, College of Chinese Medicine, China Medical University, 91 Hsueh-Shih Road, Taichung 40402, Taiwan; ^2^Graduate Institute of Medicine, College of Medicine, Kaohsiung Medical University, Kaohsiung 807, Taiwan; ^3^Department of Pathology, Kaohsiung Medical University Hospital, Kaohsiung 807, Taiwan; ^4^Department of Pathology, College of Medicine, Kaohsiung Medical University, Kaohsiung 807, Taiwan; ^5^Department of Surgery, Kaohsiung Municipal Hsiao-Kang Hospital, Kaohsiung 807, Taiwan; ^6^Department of Neurosurgery, Kaohsiung Medical University Hospital, Kaohsiung 807, Taiwan; ^7^Department of Surgery, College of Medicine, Kaohsiung Medical University, Kaohsiung 807, Taiwan; ^8^Department of Chinese Medicine, China Medical University Hospital, Taichung 40402, Taiwan; ^9^Acupuncture Research Center, China Medical University, 91 Hsueh-Shih Road, Taichung 40402, Taiwan

## Abstract

Glioblastoma multiforme is one of the most serious malignant brain tumors and is characterized by resistance to chemotherapy and radiation therapy. Recent studies suggest that autophagy may play an important role not only in the regulation of cancer development and progression but also in determining the response of cancer cells to anticancer therapy. The purpose of the present study was to assess the relationship between protein expressions of two autophagy markers, LC3B and Beclin-1, with clinical parameters in astrocytoma patients. Furthermore, the expression of CD133, a marker of the cancer stem-like cells, in astrocytoma patients was also investigated. A total of 106 thin-section slides were retrospectively collected from astrocytoma patients. LC3B, but not Beclin-1, protein expression was found to significantly correlate with resistance to radiation- or chemotherapy. In addition, high intensity of LC3B staining was predictive of poor prognosis. Furthermore, survival time of patients with high-level expression in both CD133 and LC3B was significantly shorter than those with weak expression in both CD133 and LC3B. These results suggest that astrocytoma cancer stem-like cells together with enhanced autophagy may cause resistance to radiation therapy/chemotherapy and that targeting the cancer stem-like cell in astrocytoma may offer a viable therapeutic approach.

## 1. Introduction

Astrocytoma is the most frequent brain tumor found in humans. The World Health Organization (WHO) [1] has classified astrocytomas into four grades based on the degree of malignancy. Grade I tumors are benign and slow-growing, as represented by pilocytic astrocytomas. The grade II tumors consist of relatively slow-growing diffuse astrocytomas and pilomyxoid astrocytomas. The grade III and the grade IV tumors are highly malignant and are, respectively, exemplified by anaplastic astrocytoma and glioblastoma multiforme (GBM), which is the most common and most aggressive malignant primary brain tumor in humans. Extensive efforts have been focused on identifying biomarkers that correlate with the severity of astrocytomas in order to facilitate diagnosis as well as to develop therapeutic agents for the treatment of this devastating disorder. In this regard, increased protein and/or gene expression of several biomarkers, such as cycloxygenase-2 [2], insulin-like growth factor-binding proteins [3], and epidermal growth factor receptor [4], have been shown to correlate with poor survival in astrocytoma patients. By contrast, protein and/or gene expression of* myo*-inositol [5] and N-myc downstream-regulated gene [6] have been reported to be negatively correlated with pathological grading in astrocytoma. However, these markers are rarely related to mechanisms by which the development of astrocytomas is regulated.

The cancer stem cell (CSC) theory postulates that tumors arise from a subpopulation of cells that are characterized by self-renewal, infinite proliferative potential, and multipotency and are able to initiate new tumors in vivo [7]. CSC cells mediated radio- and chemoresistance and these cells with stem-like features have been identified in glioblastoma [8]. A previous study showed that these cells express the transmembrane glycoprotein prominin-1 (CD133) (a cell-surface marker expressed on normal human neuronal stem cell) and have the ability to initiate new tumor in vivo after xenotransplantation in mice. But few data are available on the actual prognosis of CD133 expression in malignant gliomas. Glioblastoma stem cells are highly resistant to conventional chemotherapy and radiotherapy [9, 10] and the chemo-radioresistance of these cells may be responsible for the poor clinical outcome of these patients. The CSCs display strong capability of tumor resistance to TMZ. This resistance is probably contributed by the CD133+ cells with downregulation of autophagy-related proteins [11].

Autophagy constitutes the basic catabolic mechanism that involves degradation of unnecessary or dysfunctional cellular components by lysosomes [12]. Recent studies suggest that autophagy may play an important role not only in the regulation of cancer development and progression but also in determining the response of cancer cells to anticancer therapy [13–15]. Subsequent studies have also identified microtubule-associated protein 1 light chain 3 (LC3) [16–18] and Beclin-1 [19–21] as essential markers for autophagy. There are three isoforms of LC3, namely, LC3A, LC3B, and LC3C. Lines of evidence have shown that LC3B is a prognostic marker in advanced breast cancer after chemotherapy [22].

In the present study, protein expressions of LC3B and Beclin-1 in astrocytoma patients were evaluated and the results were utilized to correlate with clinical parameters. Furthermore, since cancer stem-like cells have been found to be attractive targets for novel anticancer therapies [23, 24], the expression of CD133, a marker of these cancer stem-like cells [25, 26], in astrocytoma patients was also investigated.

## 2. Material and Method

### 2.1. Astrocytoma Samples

Based on the operative notes, medical records, pathological reports, and MRI images, 218 thin section slides were retrospectively collected from astrocytoma patients who were diagnosed between 2000 and 2010 at the Neurosurgery Department of Chung-Ho Hospital, Kaohsiung, Taiwan. However samples with poor immunohistochemical staining were excluded, so were those from patients who were diagnosed by biopsies only, had incomplete medical records or no follow-up visits, or showed low quality pathological results. A total of 106 samples were finally selected for the present study. This study was approved by the Kaohsiung Medical University Hospital Review Board (KMUH-IRB-20120238).

### 2.2. Immunohistochemistry

In order to retrieve antigens for immunohistochemical staining, 3 *μ*m sections from formalin-fixed, paraffin-embedded tissue blocks were deparaffinized, rehydrated, and autoclaved at 121°C for 10 min in Target Retrieval solution (Dako, Glostrup, Denmark), pH 9.0. Endogenous peroxidase in the sections was blocked by incubating in 3% hydrogen peroxide at room temperature for 5 min. The sections were then washed in a Tris-buffered solution (Dako, K9001) and incubated with a 1 : 200 dilution of rabbit polyclonal anti-human CD133 (Biorbyt, orb18124, UK), Beclin-1 (abcam, ab16998, USA), and LC3B (SANTACRUZE, sc-16755, Europe) antibodies for 1 hr at room temperature. After washing with Tris-buffered solution, the sections were incubated with secondary antibodies conjugated with horseradish peroxidase for 30 min at room temperature. Subsequently, the slides were incubated in 3,3-diaminobenzidine for 5 min followed by Mayer's hematoxylin counterstaining for 60 sec and mounted with Entellan (product no. HX247305, Merck). The immunohistochemically stained slide sections were evaluated by an investigator blinded to the experimental procedures. The staining score of Beclin-1 and LC3B was as follows: negative: 0 (without or less 10% positive cells of tumor); weakly positive: 1 (10~30% positive cell of tumor); positive: 2 (30~70% positive cell of tumor); strongly positive: 3 (70%~100% positive cell of tumor). The staining score of CD133 was as follows: negative: 0 (if 65% of cells were stained by the antibody); weakly positive: 1 (>5–20%, including 20%, of stained cells); positive: 2 (>20–50%, including 50%, of stained cells); strongly positive: 3 (>50% of stained cells) [27]. For statistical analysis, scores of 0 and 1 were defined as low-expression group and scores of 2 and 3 were defined as high-expression group [28]. The results of protein expressions were correlated with clinical parameters such as age, gender, tumor grade, being accepted for radiation- or chemotherapy, and Karnofsky performance status scale (KPS) [29].

### 2.3. Data Analyses

Social Sciences for Windows, Version 19.0 (SPSS, Chicago, IL, USA), was used for statistical analysis. Chi-square test was performed to determine whether there was a correlation between Beclin-1 and LC3B protein expressions with a specific clinicopathological parameter. A *P* value of <0.05 was considered statistically significant. The survival rate was analyzed by the Kaplan-Meier method with log-rank test.

## 3. Results

### 3.1. Correlation between LC3B and Beclin-1 Protein Expressions with Clinical Parameters

**Figure 1:**
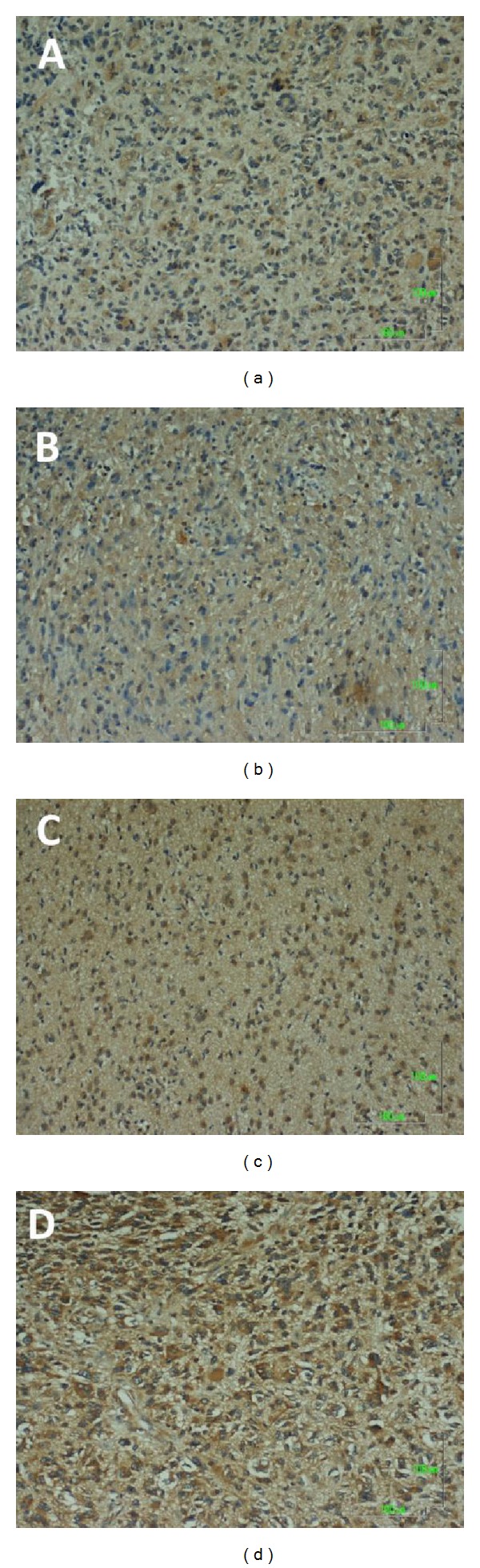
Representative immunohistochemical staining for Beclin-1 protein expression in astrocytoma sections. A: Score 0, negative or weak intensity. B: Score 1, low intensity. C: Score 2, moderate intensity. D: Score 3, high intensity. Magnification, 100x.

**Figure 2:**
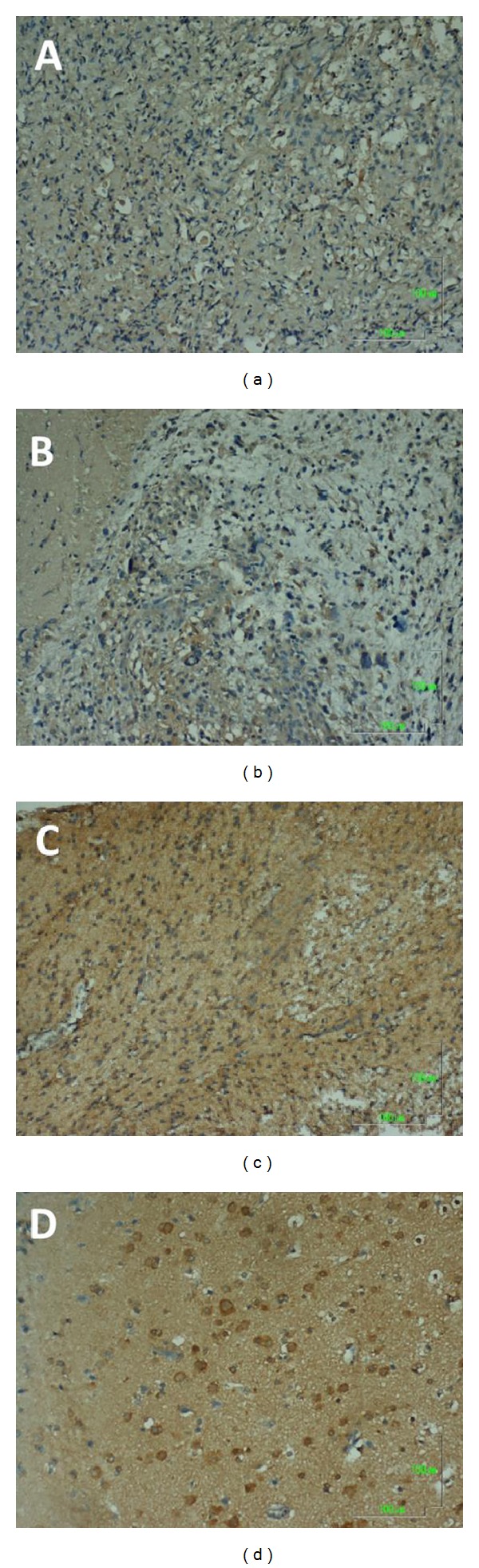
Representative immunohistochemical staining for LC3B protein expression in astrocytoma sections. A: Score 0, negative or weak intensity. B: Score 1, low intensity. C: Score 2, moderate intensity. D: Score 3, high intensity. Magnification, 100x.

**Figure 3:**
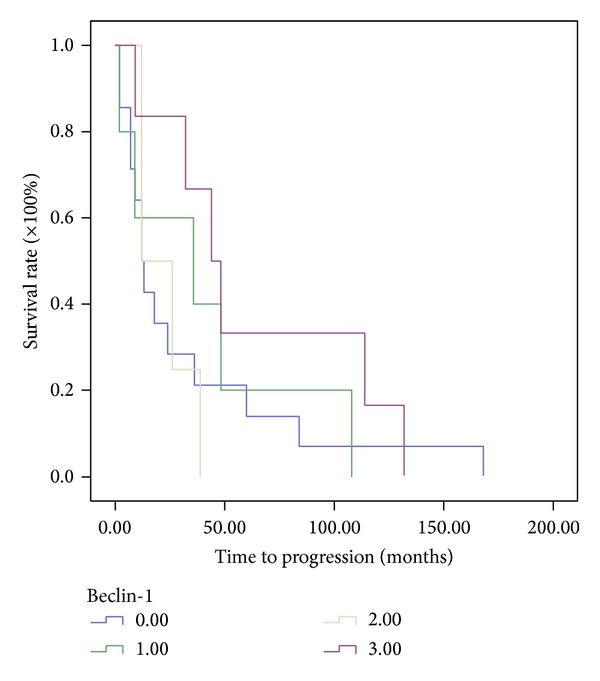
Assessment of the relationship between Beclin-1 protein expression and overall survival of astrocytoma patients using Kaplan-Meier method with log-rank test. No statistically significant correlation was found.

**Figure 4:**
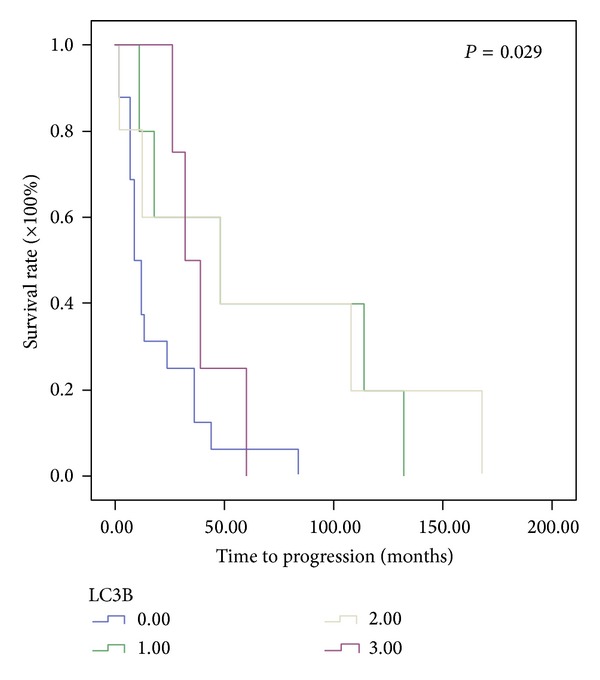
Assessment of the relationship between LC3B protein expression and overall survival of astrocytoma patients using Kaplan-Meier method with log-rank test. High intensity of LC3B immunohistochemical staining was shown to predict poor prognosis. Similar survival curve was also found with negative or weak LC3B protein expression. By contrast, low and moderate levels of LC3B expression had a significant increase in survival when compared with those of weak or high LC3B levels.

Figures 1 and 2 show representative immunochemical staining sections for Beclin-1 and LC3B, respectively, with weak, low, moderate, and high intensities. The results of immunohistochemical staining of Beclin-1 and LC3B were separately analyzed to determine the relationship of protein expression with clinicopathological parameters of astrocytoma patients, such as age, gender, tumor grade, resistance to radiation- or chemotherapy, and KPS scale. None of these parameters were significantly correlated with Beclin-1 protein expression (Table 1). LC3B protein expression was found to significantly correlate with radiation- or chemotherapy (*P* < 0.05). However, none of other clinical parameters were shown to correlate with LC3B protein expression (Table 1). Furthermore, Beclin-1 protein expression did not correlate with overall survival of the patients (Figure 3). In contrast, a high intensity in immunochemical staining of LC3B predicted poor prognosis (Figure 4). Likewise, negative or weak LC3B protein expression displayed a similar survival curve as that of high LC3B levels. The results also showed that low and moderate levels of LC3B expression had a significant increase in survival when compared with those of high LC3B levels (Figure 4).

### 3.2. Prognostic Values of CD133 and LC3B Expressions in Astrocytoma

**Figure 5:**
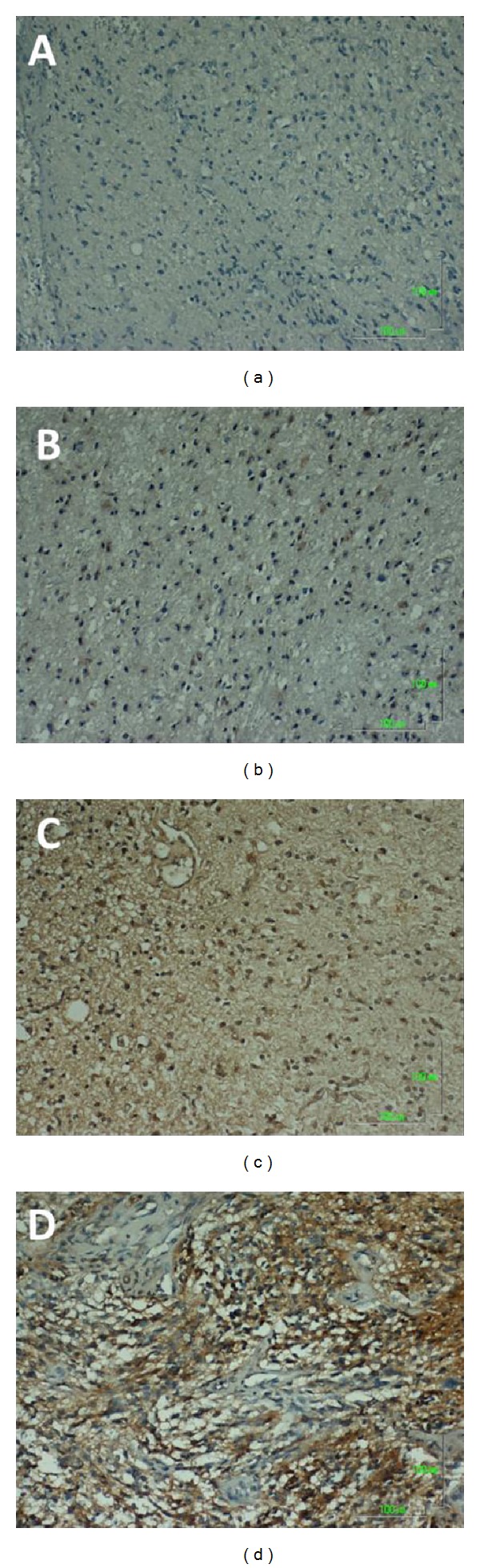
Representative immunohistochemical staining for CD133, a cancer stem-like cell marker, in astrocytoma sections. A: Score 0, negative or weak intensity. B: Score 1, low intensity. C: Score 2, moderate intensity. D: Score 3, high intensity. Magnification, 100x.

**Figure 6:**
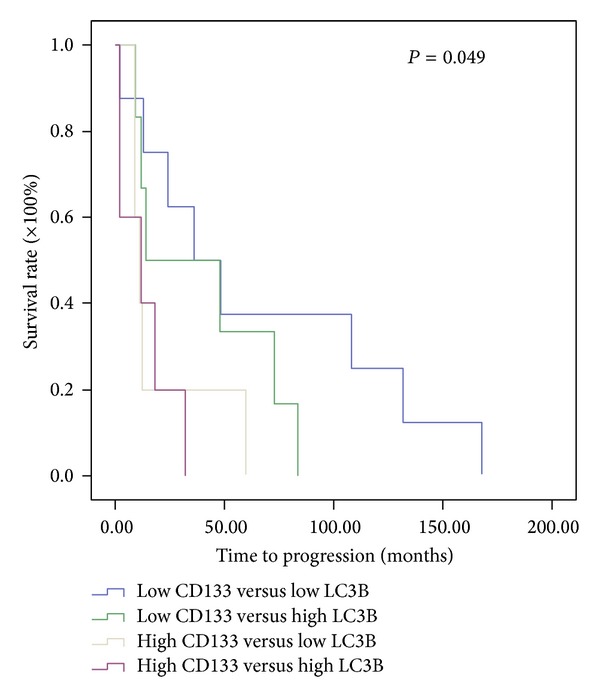
Assessment of the relationship between a combination of CD133 and LC3B protein expressions and overall survival of astrocytoma patients using Kaplan-Meier method with log-rank test. The average overall survival of patients with high-level expression in both CD133 and LC3B was 38 mon, and those with weak expression in both CD133 and LC3B was 170 mon.

A previous study has shown that cancer stem-like cells are resistant to radiation therapy or chemotherapy, and enrichment in these cells is indicative of poor prognosis [30]. Since the expression of LC3B protein was shown to correlate with resistance of radiation- and chemotherapy (Table 1), we assessed the expression of CD133, an astrocytoma cancer stem-like cell marker, together with that of LC3B in overall survival.  Figure 5 shows representative immunochemical staining sections for CD133 with weak, low, moderate, and high intensities. The overall survival of patients with high-level expression in both CD133 and LC3B was 38 mon, and those with weak expression in both CD133 and LC3B was 170 mon (Figure 6). These results showed that high-level expression of both CD133 and LC3B was indicative of poor prognosis in astrocytoma (*P* < 0.05).

## 4. Discussion

Glioblastoma multiforme (GBM), or grade IV astrocytoma, is the most frequently found class of malignant primary brain tumor and one of the most aggressive forms of cancer. As a consequence, median survival after diagnosis is usually just 12 mon [31]. Standard therapy for the management GBM includes surgical resection, focal radiotherapy, and treatment with alkylating agents such as temozolomide [32, 33]. Unfortunately, these therapeutic approaches increase the survival of GBM patients only modestly. Thus, extensive studies have focused on identifying new pathways and/or molecular markers that are predictive of poor prognosis and resistance to radiotherapy/chemotherapy in this class of patients.

There are several key findings presented in this study. Increased expression of LC3B, an autophagy marker, was found to be correlated with radiotherapy/chemotherapy as well as with poor survival in astrocytoma patients. Furthermore, increased expression of LC3B together with enhanced levels of CD133, a cancer stem-like cell marker, also correlated significantly with poor prognosis. Previous studies have shown that chemical induction of autophagy enhances chemosensitivity and radiosensitivity in papillary thyroid cancer [34]. Song et al. reported that autophagy inhibitors may make liver cancer stem cells (LCSCs) more sensitive to the tumor microenvironment and be useful in improving anticancer treatments [35]. Likewise, induction of autophagy in glioma-initiating cells by rapamycin also increased their sensitivity to radiation [36].

Autophagy constitutes the basic catabolic mechanism that involves the degradation of unnecessary or dysfunctional cellular components by lysosomes [12]. Autophagy has the ability to protect cells against metabolic stress by removing damaged or aged organelles, toxic metabolites, or intracellular pathogens [37–40]. Previous studies reported that inhibition of autophagy by 3-methyladenine (3-MA) and Atg7 siRNA enhances 5-FU induced cytotoxicity in human colorectal cancer cells [34]. Autophagy suppression also enhances the therapeutic efficacy of cisplatin and 5-FU in esophageal and colon cancer cells, respectively [41, 42].

These results drastically differ from our finding that high levels of autophagy marker LC3B led to a significant correlation with resistance to radiation- and/or chemotherapy. In our study, enhanced autophagy was a result of disease progression rather than due to chemical induction as reported by other investigators [34, 36]. In addition, the cell type used in the present study was clearly different from those utilized in other studies [34, 36] as GBM is highly resistant to chemotherapy and radiotherapy. Further investigation is needed to clarify this matter. Unexpectedly, Beclin-1, another autophagy maker, did not exhibit properties similar to those of LC3B in the current study. The reasons for this difference are not known at the present.

Huang et al. reported that the expressions of LC3B-II and Beclin-1 were reduced in GBM due to a downregulated autophagic capacity [27]. Consistent with these results, we also found that high-level expression of LC3B was associated with poor prognosis. Our results suggest that a reduction in autophagy may lead to more advanced astrocytoma although their correlation did not reach statistical significance. The role autophagy plays in tumor development is complicated [15]. It can suppress tumor development during early stages of tumorigenesis. However, autophagy can also promote further tumor development in established tumors. Our results showed that high levels of LC3B expression, or enhanced autophagy, correlated with poor prognosis are consistent with the role of autophagy as tumor promoter.

Recently, GBM cancer stem-like cells have been shown to participate in the formation of the tumor endothelium, increase in the invasiveness of the tumor, and lead to resistance to radiotherapy [43, 44] through various mechanisms. Our results showed that a high-level expression of CD133, a cancer stem-like cell marker, together with a high-level expression of LC3B, was indicative of poor prognosis in astrocytoma (Figure 6). These results suggest that astrocytoma cancer stem-like cells together with enhanced autophagy may cause resistance to radiation therapy/chemotherapy and that targeting the cancer stem-like cell in astrocytoma may offer a viable therapeutic approach.

## Figures and Tables

**Table 1:** Correlation between Beclin-1 and LC3B protein expression with clinicopathological parameters. *P* values were determined by Chi-squares analysis. KPS: Karnofsky performance status scale.

Score	LC3B					Beclin-1				
	0	1	2	3	*P*	0	1	2	3	*P*
Age					0.312					0.244
>65	16	19	14	15		9	17	18	20	
<65	12	12	17	1		7	12	13	10	
Gender					0.221					0.453
M	15	17	18	6		4	14	19	19	
F	13	14	13	10		12	15	12	11	
Tumor grade					0.563					0.312
I	2	5	3	0		0	2	4	4	
II	4	8	2	2		1	4	5	6	
III	5	4	6	2		4	5	4	4	
IV	17	14	20	12		11	18	18	16	
Radiation/chemotherapy					0.021*					0.071
Yes	8	10	24	15		8	18	19	21	
No	20	21	7	1		8	11	12	9	
KPS					0.452					0.549
>70	10	15	17	6		10	15	8	15	
<70	18	16	14	10		6	14	23	15	
